# Prediction of brain clozapine and norclozapine concentrations in humans from a scaled pharmacokinetic model for rat brain and plasma pharmacokinetics

**DOI:** 10.1186/1479-5876-12-203

**Published:** 2014-08-20

**Authors:** Claire H Li, Robert E Stratford, Nieves Velez de Mendizabal, Thomas IFH Cremers, Bruce G Pollock, Benoit H Mulsant, Gary Remington, Robert R Bies

**Affiliations:** Division of Clinical Pharmacology, Department of Medicine, Indiana University School of Medicine, 1001 W. 10th Street W7138, Indianapolis, IN 46202 USA; Indiana Clinical and Translational Sciences Institute (CTSI), Indianapolis, IN USA; Xavier University of Louisiana, New Orleans, LA USA; Department of Biomonitoring and Sensoring, Pharmacy, University of Groningen, Groningen, The Netherlands; Brains On-Line, South San Francisco, CA USA; Centre for Addiction and Mental Health, University of Toronto, Toronto, Canada

**Keywords:** Clozapine, Norclozapine, Receptor occupancy, Population pharmacokinetics, Translational modeling, NONMEM

## Abstract

**Background:**

Clozapine is highly effective in treatment-resistant schizophrenia, although, there remains significant variability in the response to this drug. To better understand this variability, the objective of this study was to predict brain extracellular fluid (ECF) concentrations and receptor occupancy of clozapine and norclozapine in human central nervous system by translating plasma and brain ECF pharmacokinetic (PK) relationships in the rat and coupling these with known human disposition of clozapine in the plasma.

**Methods:**

Unbound concentrations of clozapine and norclozapine were measured in rat brain ECF using quantitative microdialysis after subcutaneous administration of a 10 mg/kg single dose of clozapine or norclozapine. These data were linked with plasma concentrations obtained in the same rats to develop a plasma–brain ECF compartmental model. Parameters describing brain ECF disposition were then allometrically scaled and linked with published human plasma PK to predict human ECF concentrations. Subsequently, prediction of human receptor occupancy at several CNS receptors was based on an effect model that related the predicted ECF concentrations to published concentration-driven receptor occupancy parameters.

**Results:**

A one compartment model with first order absorption and elimination best described clozapine and norclozapine plasma concentrations in rats. A delay in the transfer of clozapine and norclozapine from plasma to the brain ECF compartment was captured using a transit compartment model approach. Human clozapine and norclozapine concentrations in brain ECF were simulated, and from these the median percentage of receptor occupancy of dopamine-2, serotonin-2A, muscarinic-1, alpha-1 adrenergic, alpha-2 adrenergic and histamine-1 for clozapine, and dopamine-2 for norclozapine were consistent with values reported in the literature.

**Conclusions:**

A PK model that relates clozapine and norclozapine disposition in rat plasma and brain, including blood–brain barrier transport, was developed. Using allometry and published human plasma PK, the model was successfully translated to predict clozapine and norclozapine concentrations and accordant receptor occupancy of both agents in human brain. These predicted exposure and occupancy measures at several receptors that bind clozapine may be employed to extend our understanding of clozapine’s complex behavioral effects in humans.

**Electronic supplementary material:**

The online version of this article (doi:10.1186/1479-5876-12-203) contains supplementary material, which is available to authorized users.

## Background

Schizophrenia is a debilitating disorder that affects approximately 1% of the global population without regard to race, sex or socioeconomic status [1]. Its onset occurs typically in the late-teen years or early twenties and is characterized by a high rate of morbidity and mortality. Given these high personal and societal costs, investment in research aiming at understanding the biology of the disease, its genetic components and their interplay with environmental factors, continues on many levels. Over the past 50 years, pharmacotherapeutic support has been instrumental in managing primarily the positive symptoms of schizophrenia. It hinges on suppression of a central circuitry dysfunction that can be normalized by antagonism of dopamine D2 receptors in the striatum [2]. Introduction of clozapine, the first so-called atypical antipsychotic approximately 25 years ago, represented a significant advance in our understanding of schizophrenia from a systems biology perspective in that this drug did not have the typical side effects of the first generation neuroleptics. This reduction in side effects was attributed to higher 5HT2A than D2 receptor binding [3].

However, clozapine pharmacology is not limited to D2 and 5HT2A antagonism. Albeit unintentionally, the drug binds to several other dopamine and serotonin receptor subtypes, muscarinic M1/M4 receptors, and alpha-1 adrenergic receptors with pharmacologically relevant affinity [4]. From a clinical perspective, this broad receptor coverage may account for clozapine’s unique superiority in treatment resistant schizophrenia (TRS), even amongst other atypical antipsychotics. From a research perspective, the broad receptor coverage of clozapine conceivably makes the drug a useful tool to advance our understanding of complex pharmacotherapy that incorporates multiple interacting receptor systems.

The use of positron emission tomography (PET) imaging to measure receptor occupancy of clozapine and other atypical antipsychotics in humans has been invaluable in demonstrating the importance of D2 and 5HT2A receptor antagonism contributing to the efficacy of these drugs [5–7]. However, broader application of this non-invasive technique has been limited by the lack of ligands specific for other receptors to which clozapine has affinity. In this regard, availability of other approaches that are complementary to PET imaging would be useful. One possibility is to link non-clinical measurements of clozapine disposition in the brain with clinical studies of clozapine systemic exposure using a translational PK modeling approach. Prediction of clozapine CNS exposure could then be related to its receptor binding kinetics at multiple receptors to impart a virtual predicted pharmacodynamic component to a model. This approach has been used recently to predict CNS concentrations of atomoxetine and duloxetine that were in the range of receptor affinities associated with therapeutic doses [8]. In a related manner, a population pharmacokinetic-pharmacodynamic (PK-PD) modeling approach was used to predict D2 receptor occupancy of olanzapine in humans [9], and the D2 and 5HT2A receptor occupancy of risperidone and its active metabolite paliperidone (9-OH risperidone) [5–7, 10]. These studies, as well as earlier PK-PD models applied to other CNS drugs [11, 12], provide confidence in the ability of this approach to deepen our understanding of drug action in human brain.

A recent study measured clozapine and its N-desmethyl metabolite, norclozapine, in extracellular fluid (ECF) of rat medial prefrontal cortex using quantitative microdialysis, and these results provided evidence of net efflux from brain across the blood–brain barrier (BBB) [13]. This suggests that plasma concentrations may not be a good predictor of brain concentration for clozapine or norclozapine. Therefore, prediction of clozapine exposure in the ECF of human brain using a translational PK modeling approach could be cross-validated against PET results at D2 and 5HT2A receptor occupancy in humans, and subsequently used to estimate clozapine receptor occupancy at the drug’s other receptor targets for which PET tracers do not exist. Such comprehensive PK-PD model could potentially support individualized dosing of clozapine to improve its efficacy and CNS tolerability. It would also support research aimed at discovering new approaches for the treatment of schizophrenia in its different forms.

The purpose of this study was; (1) to build a PK model that accounted for both plasma and brain concentrations measured in rats; (2) to utilize this model to predict concentrations of clozapine and norclozapine in human brain. This would allow for the prediction of expected receptor occupancy in humans.

## Methods

### Study design

A single dose of clozapine (10 mg/kg) was administered subcutaneously to four male Wistar rats with an average weight of 0.35 kg purchased from Harlan (Zeist, The Netherlands). Three days prior to administration a microdialysis guide cannula was surgically implanted in the medial prefrontal cortex; at the same time, a catheter for blood sample collection was placed in the right jugular vein and was exteriorized through an incision at the top of the head. This vascular cannulation enabled an equivalent volume of saline replacement for each blood sample. A MetaQuant probe (6 mm, cellulose membrane, BrainLink, The Netherlands) was inserted into the guide cannula 24 hours prior to drug administration to enable sampling of brain extracellular fluid (ECF). Concentrations of clozapine and its N-desmethylated metabolite, norclozapine, were measured in plasma and brain ECF by HPLC with tandem mass spectrometry in the positive ion mode as previously described [13]. For each rat, the unbound concentrations in each compartment were measured at 9 time points (0, 15, 30, 60, 90, 120, 240, 360 and 480 minutes) in plasma and 18 time points (-30, 0, 30, 60, 90, 120, 150, 180, 210, 240, 270, 300, 330, 360, 390, 420, 450 and 480 minutes) in brain. A single dose of norclozapine (10 mg/kg) was also administered subcutaneously to another five male Wistar rats with an average weight of 0.34 kg bought from the same Harlan laboratories. Concentrations were measured in plasma and brain ECF, and the same time points were used as those specified for clozapine.

### Model development

Different model structures were initially evaluated using the system dynamics software VENSIM (Ventana Systems, Inc., MA, US). Thereafter a population approach was used to describe the pharmacokinetics of clozapine and norclozapine. Population PK parameters were estimated using a nonlinear mixed effect modeling approach, as implemented in NONMEM version 7.2 (Icon Development Solutions, Hanover, Maryland) using Wings for NONMEM version 7 [14]. The first-order conditional estimation method (FOCE) with interaction was used to estimate the structural PK parameters and the random effects parameters.

Model development was started with an assessment of clozapine PK in plasma. One and two compartment models with first order absorption for clozapine in plasma were tested. A peripheral compartment structure was subsequently implemented to represent the brain extracellular fluid concentrations. The transfer characteristics of clozapine between the plasma and the brain compartment were evaluated using an intercompartmental clearance, CLin/CLout, as well as incorporating delay functions [15]. These delay functions included a lag time and transit compartment approaches. Once the structural model for clozapine was established, the plasma compartment of norclozapine was integrated and then connected to the brain compartment. The same strategy was utilized in building the structural model for norclozapine concentrations that were measured following norclozapine administration. Clozapine and norclozapine concentration measurements were then combined from the 9 rats and modeled simultaneously in the final structural model. The volume of distribution of clozapine and norclozapine in brain were tested with and without fixing this parameter to a literature reported value [16]. A parallel metabolic pathway from the extravascular space was also explored.

Between-animal variability (BAV) for PK parameters was assumed to be log-normally distributed and evaluated using an exponential model *P*_*i*_ = *P*_*TV*_*x e*^*ηp*^ where *P*_*i*_ is the parameter estimate for the i^th^ animal, and *P*_*TV*_ is the typical parameter value at the population level. The variability between i^th^ individual and population parameter values was described by *η*_*p*_, which was identically distributed with mean equal to 0 and variance, ω_*η*_^2^ [17]. A combined additive and proportional model was first used to describe the intra-animal variability. If one of the elements of the model was found to be negligible and not significant, it was then removed from the residual error model. Residual error parameters were assumed to be normally distributed with mean equal to 0 and variance, σ^2^.

### Model selection and evaluation

Model evaluation was based on a likelihood ratio test using the objective function value (OFV) provided by NONMEM. The minimum OFV returned by NONMEM is approximately equal to -2 × log likelihood (-2LL) and served as a guide during model design. A decrease in -2LL of 6.63 points for 1 degree of freedom was regarded as a significant model improvement, corresponding to a p value of 0.01 for nested models. The final model was further examined using goodness-of-fit plots generated using R version 2.13 based on the conditional weighted residuals distribution and the predicted versus observed concentrations at both the population and individual levels. Furthermore, the final pharmacokinetic model was also evaluated using a visual predictive check (VPC), and the uncertainty on each parameter was determined using a non-parametric bootstrap sampling with replacement 1000 times from the original dataset.

### Human translation and expected receptor occupancy

After the pharmacokinetic model of clozapine in rat was finalized, the PK model framework was adapted by scaling PK parameters with allometric principles to predict human concentrations in brain. The following exponents were utilized scaling body weight to: clearance 0.75; volume of distribution 1; and first order rate constants 0.25 [18]. A 50% of conversion from clozapine to norclozapine in humans was assumed in the model based on prior reports [19–21], and this was implemented in the simulated model assuming CL_clo_/F is equal to CL_clo-p_/F. Model performance was evaluated by comparing model simulated plasma concentrations to published human clozapine plasma concentrations [22] at steady state following 200, 300 and 400 mg daily doses. The published human clozapine data were reported as total concentrations, and these concentrations were converted to free concentration using 3% unbound fraction [23] prior to the comparison. After model validation, the simulated human clozapine and norclozapine concentrations were used to calculate the expected human receptor occupancy for the following receptors: dopamine 2 (D2); serotonin 2A (5-HT2A); muscarinic-1 (M1); alpha-1 adrenergic (α1); alpha-2 adrenergic (α2); and histamine-1 (H1) using published equilibrium dissociation constants (K_d_) for clozapine [24–26] and norclozapine (D2 only) [27].

## Results

### Rat population pharmacokinetics

**Figure 1:**
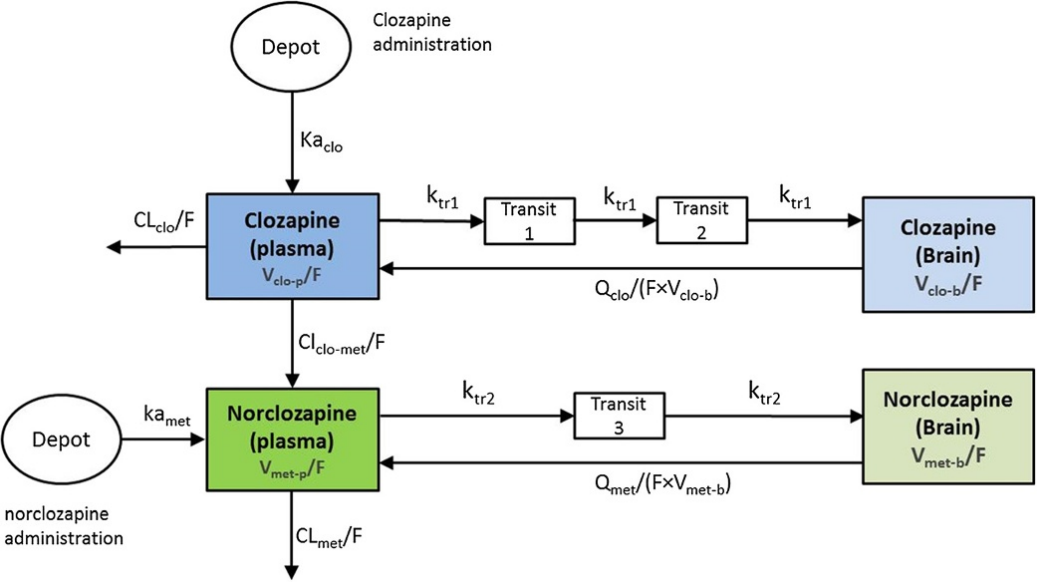
**Compartmental representation of clozapine and norclozpaine pharmacokinetics.** Two compartments in blue represented the plasma and brain compartment of clozapine. Two compartments in green represented the plasma and brain compartment of norclozapine. Clo (Clozapine): CL_clo_/F (L/min) = apparent clearance of clozapine; V_clo-p_/F (L) = apparent volume of distribution of clozapine in plasma; Ka_clo_ (1/min) = absorption rate of clozapine; Q_clo_/F. (L/min) = apparent intercompartmental clearance of clozapine; V_clo-b_ /F (L) = apparent volume of distribution of clozapine in brain; Ktr1 (1/min) = transit rate constant of clozapine. Met (Norclozapine): CL_clo-met_/F (L/min) = apparent clearance of clozapine to norclozapine; CL_met_ /F (L/min) = apparent clearance of norclozapine; V_met-p_/F (L) = apparent volume of distribution of norclozapine in plasma; Ka_met_ (1/min) = absorption rate of norclozapine; Q_met_/F (L/min) = apparent intercompartmental clearance of norclozapine; V_met-b_/F (L) = apparent volume of distribution of norclozapine in brain; Ktr2 (1/min) = transit rate constant of norclozapine.

A two-compartment model with first order absorption best described clozapine pharmacokinetics in rats using a central compartment for plasma concentrations and a peripheral compartment for brain concentrations. Between plasma and brain, an apparent delay in the distribution of clozapine was identified. Several structural models were tested to capture the observed delay. A transit compartment model with two compartments best described flow from plasma to brain, and inter-compartment clearance described the return from brain to plasma (Figure 1). Population pharmacokinetic estimates are given in Table 1. Norclozapine exposures in plasma and brain following clozapine administration were adequately described using a similar structure, but with one fewer transit compartment (K_tr2_), which was estimated to be approximately 40% of the clozapine value. The volume of distribution of clozapine (V_clo-p_/F) and norclozapine in brain (V_met-p_/F) were fixed to the previously estimated values in the final model. Although a significant reduction in the OFV was observed when both parameters were estimated, they were estimated with very poor precision. The elimination of clozapine converted to norclozapine was CL_clo-met_ at 0.055 L/min, which is approximately 10% of total clozapine systemic clearance. The NONMEM control stream with the selected model is also included in the supplementary material (Additional file 1).

**Table 1:** **Parameter estimates of final population pharmacokinetic model**

			Bootstrap analysis Median [5-95th percentiles]	
Parameters	Estimates (RSE%)	BAV (RSE%)	Estimates	BAV
Clozapine				
CL_clo_/F (L/min)	0.5(20.3)		0.463[0.358-0.661]	
V_clo-p_/F (L)	19.4(40.3)		18.9[5.43-31.59]	
Ka_clo_ (1/min)	0.00801(14.7)		0.00815[0.0051-0.0095]	
Q_clo_/F (L/min)	2.01(40.8)	0.193(33.6)	2.08[0.58-3.43]	0.2[0.07-0.29]
V_clo-b_/F (L)	0.214 FIXED			
Ktr1 (1/min)	0.0125(9.4)	0.05 (48.9)	0.0129[0.011-0.015]	0.047[0.0033-0.069]
F_clo_	1 FIXED	0.259(37.1)		0.243[0.062-0.35]
Norclozapine				
Cl_clo-met_/F (L/min)	0.055(24.8)		0.0584[0.04-0.0855]	
CL_met_/F (L/min)	0.419(18.8)	0.111(74.8)	0.43[0.33-0.61]	0.08[0.0038-0.18]
V_met-p_/F (L)	2.95(38.6)	0.168(51.1)	3.02[1.91-5.55]	0.149[0.000047-0.27]
Ka_met_ (1/min)	0.00277(31)	0.371(44.2)	0.00296[0.0015-0.0047]	0.319[0.065-0.54]
Q_met_/F (L/min)	0.388(45)		0.386[0.229-0.768]	
V_met-b_/F (L)	0.25 FIXED			
Ktr2 (1/min)	0.00517(12.3)		0.00521[0.0045-0.0064]	
F_met_	1 FIXED			
Residual error (proportional)				
Parent-plasma	0.109(70)		0.084[0.029-0.19]	
Parent-brain	0.0367(27)		0.0371[0.023-0.055]	
Metabolite-plasma	0.0762(24.1)		0.0714[0.051-0.11]	
Metabolite-brain	0.014(18)		0.0133[0.0095-0.017]	

Clo (Clozapine): CL_clo_/F (L/min) = apparent clearance of clozapine; V_clo-p_/F (L) = apparent volume of distribution of clozapine in plasma; Ka_clo_ (1/min) = absorption rate of clozapine; Q_clo_/F (L/min) = apparent intercompartmental clearance of clozapine;

V_clo-b_/F (L) = apparent volume of distribution of clozapine in brain; Ktr1 (1/min) = transit rate constant of clozapine.

Met (Norclozapine): CL_clo-met_/F (L/min) = apparent clearance of clozapine to norclozapine; CL_met_/F (L/min) = apparent clearance of norclozapine; V_met-p_/F (L) = apparent volume of distribution of norclozapine in plasma; Ka_met_ (1/min) = absorption rate of norclozapine; Q_met_/F (L/min) = apparent intercompartmental clearance of norclozapine; V_met-b_/F (L) = apparent volume of distribution of norclozapine in brain; Ktr2 (1/min) = transit rate constant of norclozapine.

BAV = between animal variability.

RSE% = percent relative standard error.

**Figure 2:**
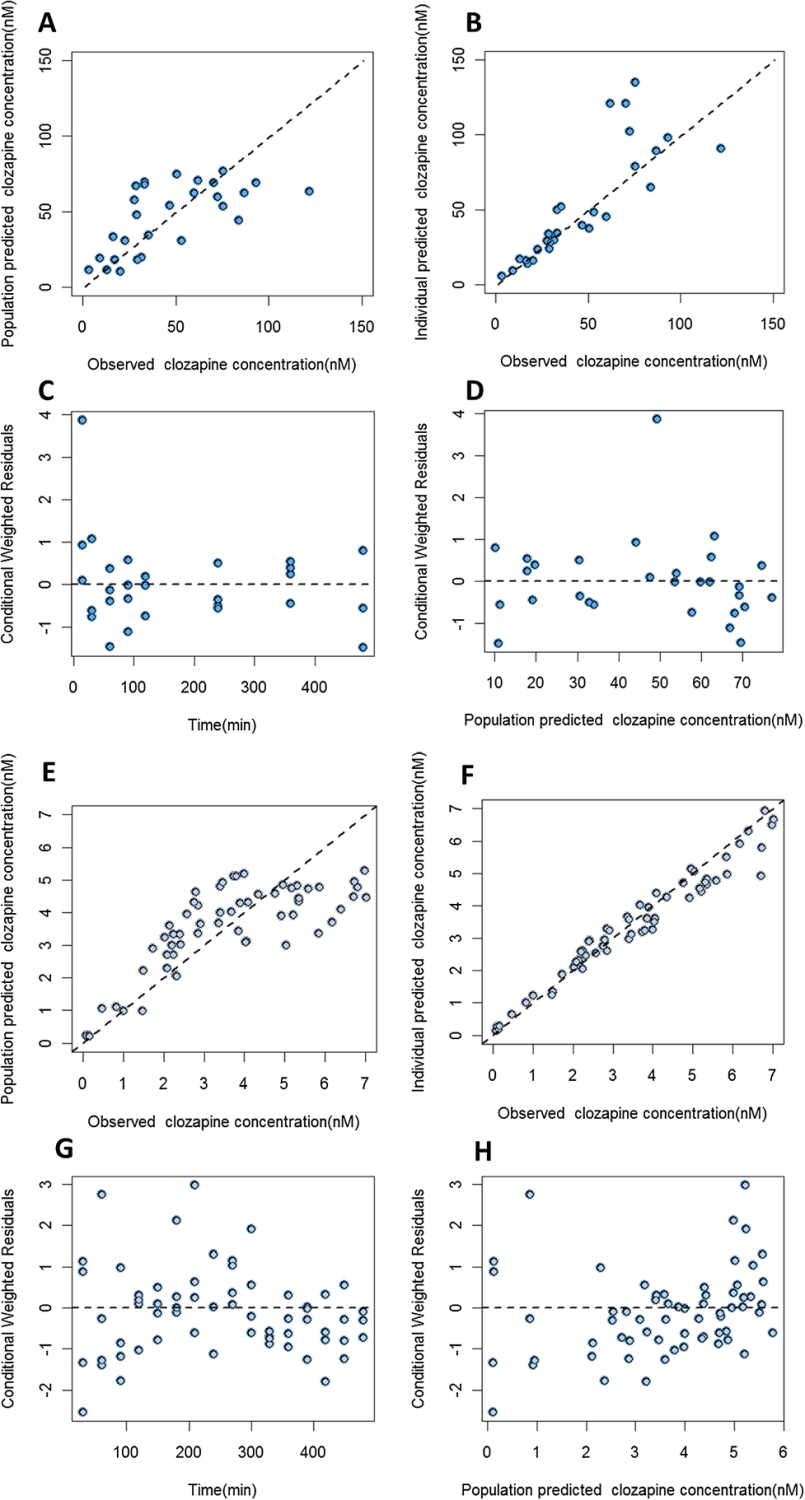
**Model diagnostic plots for clozapine. A** and **E**: population prediction vs. observation plots for clozapine in plasma and brain ECF, respectively, solid line is line of identity. **B** and **F**: individual prediction vs. observation plots for clozapine in plasma and brain ECF, respectively. **C** and **G**: conditional weighted residuals vs. time for clozapine in plasma and brain ECF, respectively. **D** and **H**: conditional weighted residuals vs. population prediction for clozapine in plasma and brain ECF, respectively.

**Figure 3:**
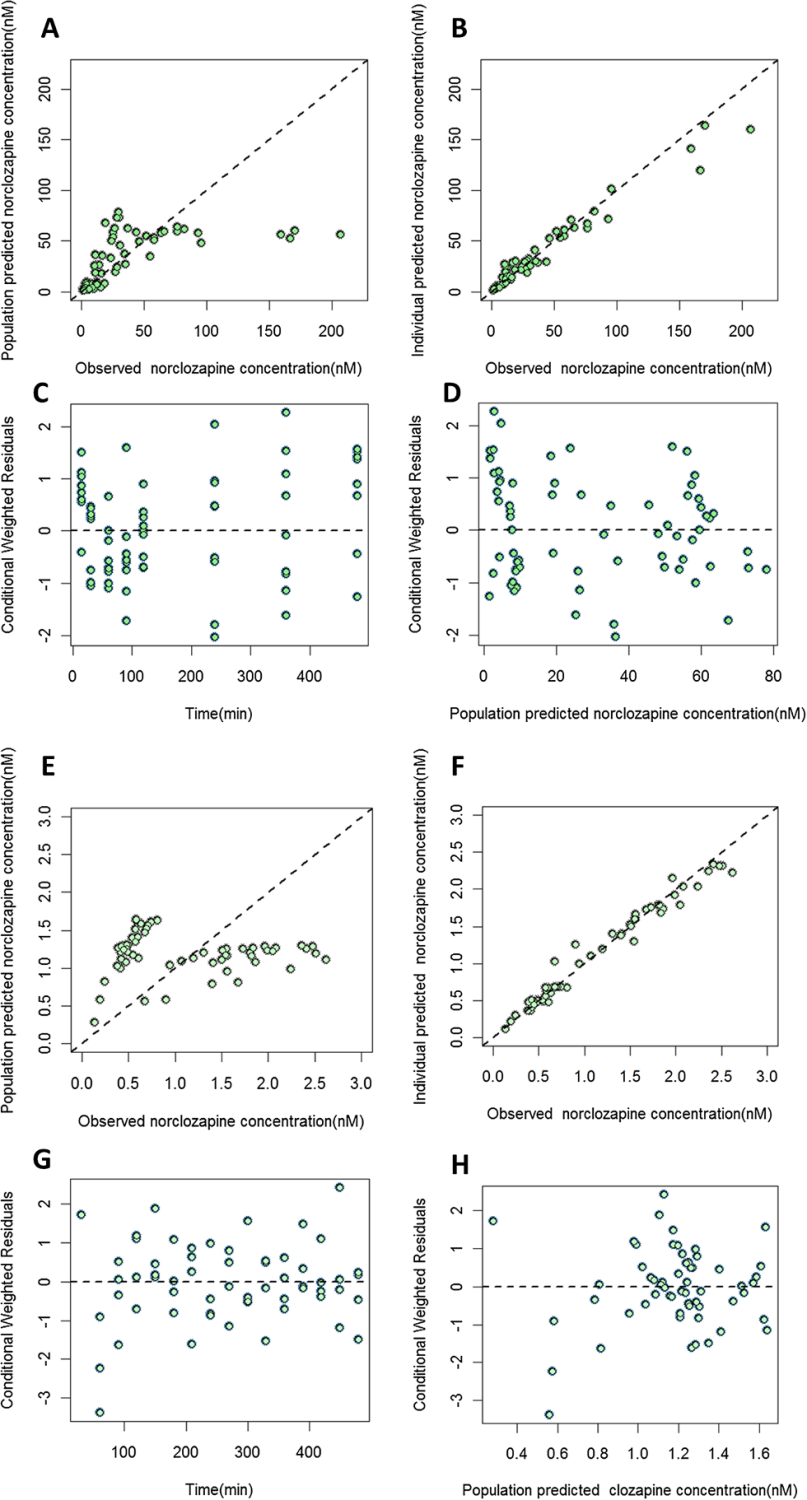
**Model diagnostic plots for norclozapine. A** and **E**: population prediction vs. observation plots for norclozapine in plasma and brain ECF, respectively, dashed line is the line of identity. **B** and **F**: individual prediction vs. observation plots for norclozapine in plasma and brain ECF, respectively. **C** and **G**: conditional weighted residuals vs. time for norclozapine in plasma and brain ECF, respectively. **D** and **H**: conditional weighted residuals vs. population prediction for norclozapine in plasma and brain ECF, respectively.

**Figure 4:**
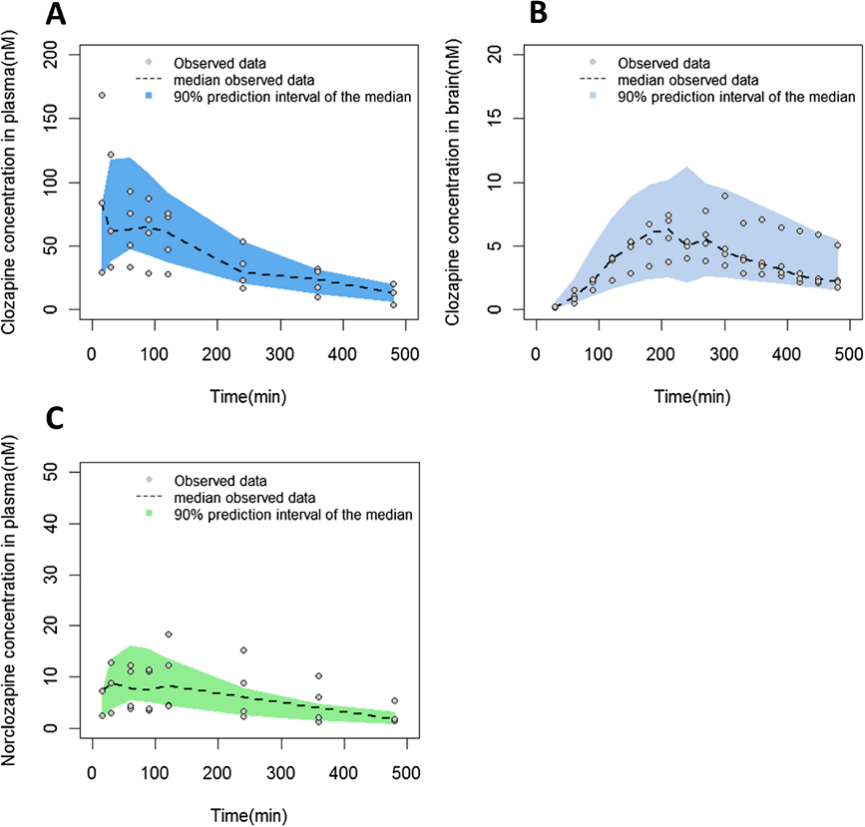
**Visual predictive checks of clozapine in plasma and brain and norclozapine in plasma.** Visual predictive check of clozapine concentrations in plasma **(A)** and brain **(B)**, and norclozapine concentrations in plasma **(C)** following a 10 mg/kg subcutaneous dose of clozapine. Norclozapine concentrations in brain following a 10 mg/kg subcutaneous dose of clozapine were not measureable. Dashed line is the median of observed concentrations, the shape represents the 90% predicted interval of the median, and the dots represent the observed concentrations.

**Figure 5:**
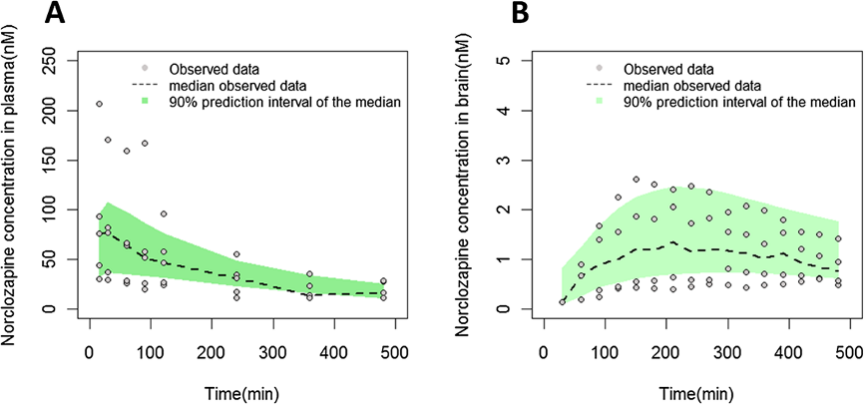
**Visual predictive checks of norclozapine in plasma and brain.** Visual predictive check of norclozapine concentrations in plasma **(A)** and brain **(B)** following a 10 mg/kg subcutaneous dose of norclozapine. Dashed line is the median of observed concentrations, the shape represents the 90% predicted interval of the median, and the dots represent the observed concentrations.

Figures 2 and 3 show the goodness of fit plots of the final model for the parent drug and metabolite in plasma and brain respectively. Population and individual predictions as well as the conditional weighted residuals distribution are shown in these figures. In the case of norclozapine, imprecision in population predicted plasma concentrations was evident and is attributed to the inter-animal variability observed in the context of the limited number of animals available to support these predictions. The majority of the fixed effects were estimated with less than 40% relative standard error (Table 1). BAV was estimated for several of the structural parameters and ranged from 5% (K_tr1_) to 75% (Cl_met_). Residual variability for clozapine in plasma and brain were 10.9% and 3.7%, respectively, and residual variability of norclozapine in plasma and brain were 7.6% and 1.4%, respectively. VPC results are shown in Figures 4 and 5. The observed median (dashed black lines) concentrations were adequately captured by the corresponding simulation based 90% predicted intervals of median concentrations for clozapine and norclozapine (shaped areas). Median, 5^th^ and 95^th^ percentiles of the parameters derived from the bootstrap analysis of 1000 replicates are shown in Table 1.

### Human translation

**Figure 6:**
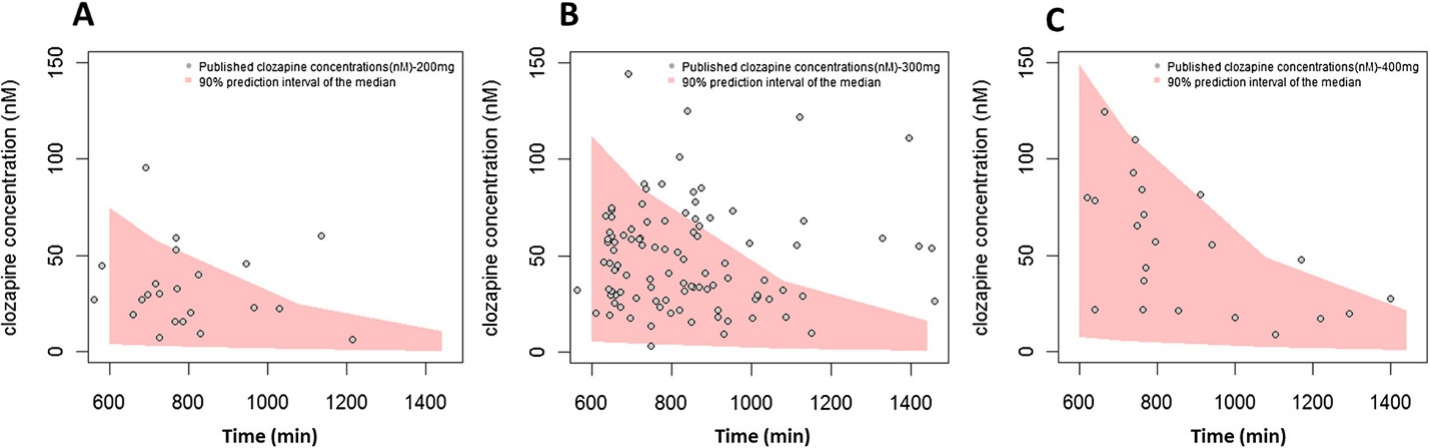
**Simulated clozapine human unbound concentrations vs. published human concentrations at steady state in plasma with 200, 300 and 400 mg OID from 10 to 24 hours.** The shape represents the 90% predicted interval of the median and the dots represent the observed data.

**Figure 7:**
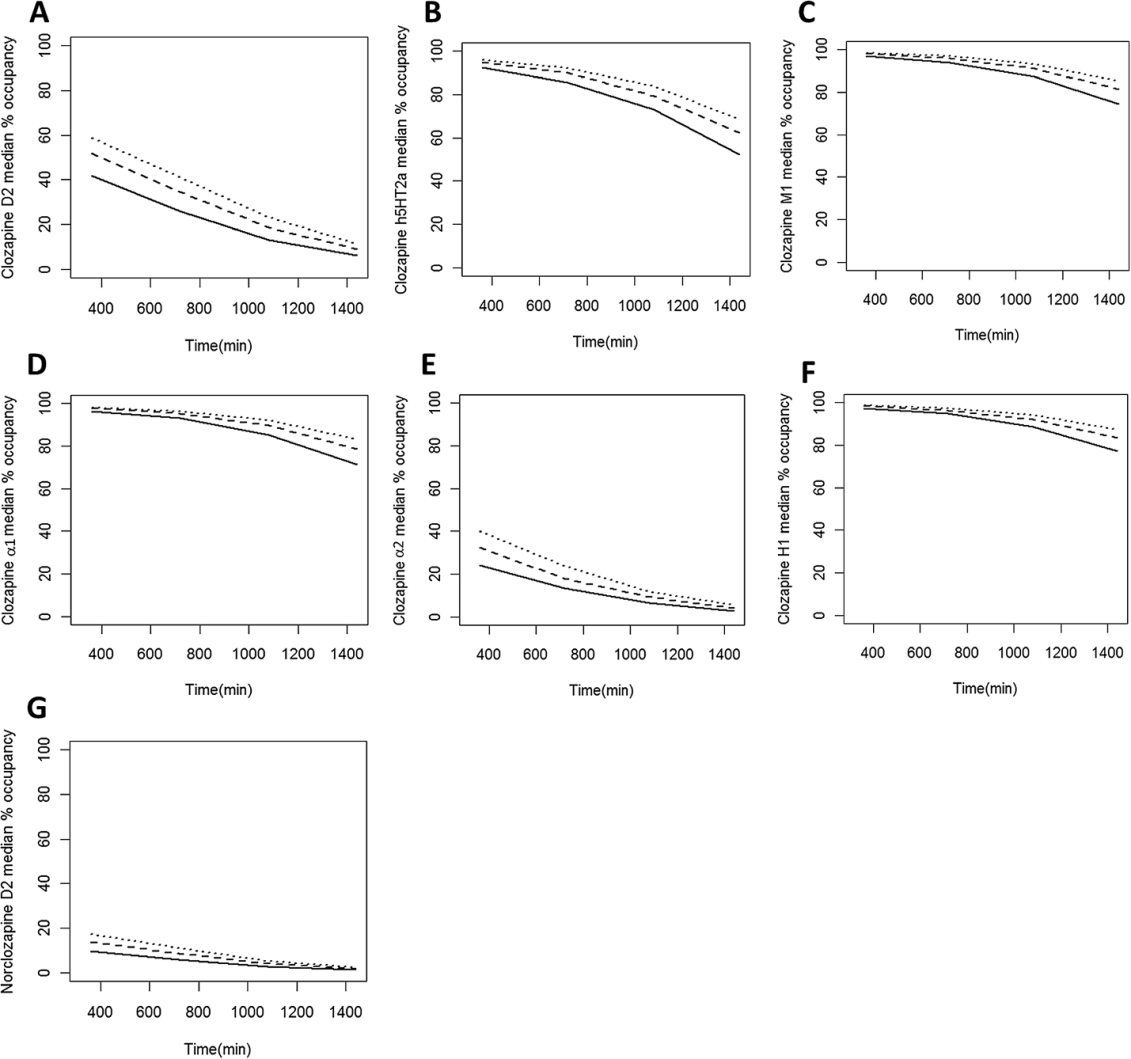
**The predicted median percentage of receptor occupancy of D2, 5-HT2A, M1, α1, α2 and H1 for clozapine and D2 for norclozapine.** The predicted median percentage of receptor occupancy of D2, 5-HT2A, M1, α1, α2 and H1 for clozapine were shown in **A** to **F**, respectively, and predicted median percentage of receptor occupancy of D2 for norclozapine was shown in **G** between 6 to 24 hour after dose. The solid line, dash line and dot line represent the predicted median percentage of receptor occupancy following 200, 300 and 400 mg daily doses, respectively.

Simulated unbound clozapine concentrations in plasma from 12 to 24 hours after administration were compared with published human data and are shown in Figures 6A, 6B and 6C for 3 doses (200, 300 and 400 mg/day). Predicted occupancy of D2, 5-HT2A, M1, α1, α2 and H1 receptors were calculated using simulated unbound clozapine and norclozapine (D2 only) brain concentrations for these three daily doses. For clozapine, the predicted median percentage of receptor occupancy of D2 ranged from 6-42%, 9-52% and 11-59% for the 200, 300 and 400 mg daily doses, respectively, across the inter-dose time interval. The median percentage of 5-HT2A receptor occupancy decreased from 93% to 52%, 95% to 62% and 96% to 69% from 6 to 24 hours after 200, 300 and 400 mg daily doses, respectively. For M1, α1 and H1 receptors, occupancies ranged from 74 to 99% across the dosage interval. In addition, the median percentage occupancy of α2 receptors was predicted to be in the range of 3-40% across the dosage interval. For norclozapine, the predicted median percentage of receptor occupancy of D2 ranged from 1.1-17.3% across the dosage interval. Receptor occupancy results across the dosage interval are summarized in Figure 7.

## Discussion

The model presented represents a unique PK model developed from directly measured concentrations of clozapine and norclozapine in rat plasma and brain ECF. A multiple transit compartment model was used to account for a delay in the transport of clozapine and norclozapine from plasma to brain across the rat blood brain barrier. Some evidence suggests that Pgp may be involved in the process of clozapine transport [13, 28] across the blood brain barrier. The need to incorporate transit compartments in the present model is consistent with a Pgp role in clozapine transport across this barrier. Using an animal model, drug exposure can be measured by microdialysis at the target site. Based on a previously published non-compartmental analysis, the ratio of AUC between parent and metabolite in the rat indicated that only about 10% of parent drug was eliminated through metabolism [29]. This is consistent with the ratio of norclozapine to clozapine clearance (Cl_clo-met_ = 9.91% of Cl_clo_). As the results revealed, even with relatively rich sampling profiles, the uncertainty of some parameters, in particular of between-animal variability, was large likely because of the small number of animals in this study.

Simulated human plasma concentrations were based on previously published human plasma concentration data [22]. The unbound plasma concentrations at steady state after a range of doses overlapped with published data corrected for the unbound fraction of clozapine (3%) [23]. Subsequently, plasma exposures were linked to the plasma – brain structural PK parameters, using allometric scaling, that described clozapine and norclozapine transport between plasma and brain in the rat to ultimately predict human brain ECF exposure. This population pharmacokinetic approach, based on a transit compartmental approach as opposed to explicit assumption of a Pgp role and its associated interspecies scaling, enabled translational representation of the system across species to predict human brain ECF concentrations. As an atypical antipsychotic drug, clozapine targets D2 receptors as well as acts as an agonist or antagonist at several other receptors found in the CNS. In order to get a more complete profile of PK-PD linkage, percentage receptor occupancy of each receptor was calculated from 6 to 24 hour after three dosage levels. Our results show that the median percentage D2 receptor occupancy was in a range of 42% to 59% 6 hours after administration of a daily dose of 200–400 mg. This range is congruent with the 33% to 67% range reported by Nordström et al. [30], and agrees with the widely recognized understanding of low D2 receptor occupancy of therapeutic doses of clozapine relative to those obtained with therapeutic doses of other antipsychotics (first and second generation). In addition to D2, percent 5-HT2a receptor occupancy also overlapped with the results of Nordström et al.

The proposed PK model thus demonstrated the ability to extrapolate human systemic exposure to predict clozapine brain concentrations and associated receptor occupancy profiles in humans at clinically relevant doses. In addition, the model simultaneously captured parent and metabolite in the system, which is relevant since norclozapine also has activity at multiple receptors [31]. However, the model can be improved in the precision of the PK parameter estimates by increasing the sample size. With this limitation taken into consideration, the model framework reported shows promise in predicting clozapine receptor occupancy at multiple receptors in human CNS, which can then be probed as a correlate to response and/or toxicity.

## Conclusions

We demonstrated that a translational PK modeling approach was able to predict clozapine and norclozapine CNS exposures in humans, and these CNS exposures could then be related to their receptor binding kinetics at multiple receptors. Such a modeling approach could be foundational to the design of comprehensive PK-PD models and extend our understanding of clozapine’s complex behavioral effects in humans.

## Electronic supplementary material

Additional file 1: **NONMEM control stream.** (DOCX 13 KB)

## Abbreviations

ECF: Extracellular fluid
CNS: Central nervous system
PK: Pharmacokinetic
PD: Pharmacodynamics
PET: Positron emission tomography
BBB: Blood–brain barrier
FOCE: First order conditional estimation method
OFV: Objective function value
TRS: Treatment resistant schizophrenia.
