# Supplementary material for: Prediction of brain clozapine and norclozapine concentrations in humans from a scaled pharmacokinetic model for rat brain and plasma pharmacokinetics
Source: J Transl Med. 2014 Aug 20;12:203. doi: 10.1186/1479-5876-12-203 (PMC4261612; doi:10.1186/1479-5876-12-203)
Supplement: Supplementary file 1 — Additional file 1: NONMEM control stream. (DOCX 13 KB) [file 12967_2014_1930_MOESM1_ESM.docx]

...

$SUBS ADVAN13 TOL=6

$MODEL

COMP(DEPOT) ; ORAL DOSE OF CLOZAPINE

COMP(CENTRAL) ; PLASMA COMPARTMENT FOR CLOZAPINE

COMP(PER) ; BRAIN COMPARTMENT FOR CLOZAPINE

COMP(TRANSIT1) ; TRANSIT COMPARMENT 1 FOR CLOZAPINE

COMP(TRANSIT2) ; TRANSIT COMPARTMENT 2 FOR CLOZAPINE

COMP(MET) ; PLASMA COMPARTMENT FOR NORCLOZAPINE

COMP(DEPOT2) ; ORAL DOSE OF NORCLOZAPINE

COMP(PER2) ; BRAIN COMPARTMENT FOR NORCLOZAPINE

COMP(TRANSIT3) ; TRANSIT COMPARTMENT 1 FOR NORCLOZAPINE

$PK

CLclo=THETA(1)

CLclo-met=THETA(2)

Vclo-p=THETA(3)

KAclo=THETA(4)

S2=Vclo-p

Vclo-b=THETA(5)

Qclo=THETA(6)*EXP(ETA(1))

Fclo=THETA(7)*EXP(ETA(2))

KTR1=THETA(8)*EXP(ETA(3))

CLmet-p=THETA(9)*EXP(ETA(4))

KAmet=THETA(10)*EXP(ETA(5))

Vmet-p=THETA(11)*EXP(ETA(6))

Vmet-b=THETA(12)

Qmet=THETA(13)

KTR2=THETA(14)

Fmet=THETA(15)

A_0(1)=0

A_0(2)=0

A_0(3)=0

A_0(4)=0

A_0(5)=0

A_0(6)=0

A_0(7)=0

A_0(8)=0

A_0(9)=0

;------------------------------------------------------------------------------------------

$DES

;----------------------PK ODES-------------------------------------------------------------

DADT(1)=-KAclo*A(1)

DADT(2)=KAclo*A(1)-(CLclo-met/Vclo-p)*A(2)-(CLclo/Vclo-p)*A(2)+(Qclo/Vclo-p)*A(3)-KTR1*A(2)

DADT(3)=KTR1*A(5)-(Qclo/Vclo-b)*A(3)

DADT(4)=KTR1*A(2)-KTR1*A(4)

DADT(5)=KTR1*A(4)-KTR1*A(5)

DADT(6)=(CLclo-met/Vclo-p)*A(2)-(CLmet/Vmet-p)*A(6)+KAmet*A(7)+(Qmet/Vmet-b)*A(8)-KTR2*A(6)

DADT(7)=-KAmet*A(7)

DADT(8)=KTR2*A(9)-(Qmet/Vmet-b)*A(8)

DADT(9)=KTR2*A(6)-KTR2*A(9)

...
